# Deterring Drunk Driving: Why Some States Go Further Than Others in Policy Innovation

**DOI:** 10.3390/ijerph16101749

**Published:** 2019-05-17

**Authors:** Suk Joon Hwang, Frances Berry

**Affiliations:** 1 Graduate School of Public Administration, Seoul National University, Seoul 08826, Korea; 2 Askew School of Public Administration and Policy, Florida State University, Tallahassee, FL 32306, USA; fberry@fsu.edu

**Keywords:** state innovativeness, bandwagon effect, comprehensiveness of state policy adoptions, DUI (Driving under Influence)

## Abstract

Policy innovation and diffusion studies have, since 1990, generally focused on a specific policy over time. Yet, few studies have considered if and why states adopt related multiple policies—a package of reforms—in a policy area. Are more innovative states in DUI policy likely to adopt a comprehensive set of policies or use them as substitutes for each other? In this study, we assess how overall state innovativeness relates to the adoption of sixteen DUI (Driving Under the Influence) laws. We find that state innovativeness in traffic safety policies (but not overall policy innovativeness), organizational size, and professionalism of a state highway department increase the likelihood that a state will adopt a more comprehensive bundle of DUI laws. Furthermore, we also test whether institutional or competitive bandwagon effects are found across this policy area and demonstrate that national institutional bandwagon effects are an important factor related to the increased comprehensiveness of state adoption of DUI policies.

## 1. Introduction 

According to the National Highway Traffic Safety Administration, over 426,547 people were killed in traffic crashes on American highways in the ten years from 2002 to 2012 [1]. About one-third of these fatalities resulted from DUI (Driving Under the Influence) crashes (even though DUI stands for the crime cases involving a driver sufficiently impaired by alcohol, drugs, or a combination of these, DUI is the most widely-used acronym for drunk driving cases. Thus, we use DUI as the abbreviation of “alcohol-impaired driving” in the entire study). Even though the alcohol-impaired driving fatalities gradually declined to 10,076 in 2013 from 13,582 in 2005, drunk driving is still a serious problem for public health. Almost 30 people a day in the United States are killed in crashes involving at least one alcohol-impaired driver (an alcohol-impaired driver is defined as having a blood alcohol concentration (BAC) of 0.08 or higher by the NHTSA. A driver’s BAC level is determined by the grams of alcohol per deciliter of blood (g/dL); a BAC of 0.08 means 8 parts of alcohol per 10,000 parts of blood. A BAC of 0.10 had been the general legal limit of DUI in most states until 1992. Mounce and Pendleton [2] showed that drivers at 0.08 BAC are significantly impaired with regard to critical driving tasks). To address this societal problem, states have adopted a number of policies for deterring alcohol-impaired driving. However, penalties and specific laws adopted by each state vary substantially. 

Until now, mainstream policy innovation and diffusion studies have focused on a single reform over time (with several exceptions, such as Boushey, 2010; Boehmke and Skinner, 2012; Nicholson-Crotty Sean, C., et al., 2014 [3,4,5].) However, in practice, a state often adopts a “package” of reforms to better achieve the policy goal. While some states adopted only a few reforms to discourage or punish drunk driving, others adopted the entire package of DUI laws introduced for drunk driving prevention in the last twenty years. Thus, this article builds on the idea that another way to examine innovativeness is to look at a broad policy area and examine the extent of states’ adoptions among possible laws. We take this approach by asking: why do some states go further than others in deterring drunk driving?

This study also contributes to the literature on state diffusion of policies by testing whether a “bandwagon” effect exists, influencing the diffusion of state DUI laws. Abrahamson and Rosenkopf’s definition of “bandwagon” diffusion can be summarized as a process whereby a policy or management fad spreads across different systems because it becomes popular, rather than being based on clear results and rational efficiency [6]. Both the institutional and competitive bandwagon effects, found in a limited number of management and policy studies [7,8,9], will be tested in our multivariate models.

To explain why some states go further than others in controlling drunk driving in their jurisdictions, this article proceeds in the following manner. First, the current status of alcohol impaired driving is briefly reviewed to understand the severity of drunk driving and what kinds of policies have been adopted by states to decrease it. Next, theoretical arguments about why some states’ set of policy adoptions are more comprehensive will be provided with testable hypotheses. Then, the data and methodology used in this study are described. Finally, the significant insights and limitations of this study are discussed based on the results.

## 2. States’ Adoptions of the DUI Laws as Policy Innovations

To decrease the social losses caused by drunk driving, states have adopted a large number of DUI policies; we utilize sixteen of the most prevalent and evidence-based policies in this analysis. Even though some DUI policies have been adopted by all states, such as MLDA 21 (21-year-old minimum legal drinking age), Implied Consent Law, etc., there have been substantial differences in how many of these sixteen policies each state has adopted to reduce and control drunk driving. In our narrative, we will refer to the extent to which states have adopted all sixteen laws, with all sixteen laws being considered a comprehensive approach to addressing drunk driving.

Most innovation and diffusion studies have focused on whether a state adopted a certain policy by the end of the time period, and how early a state adopted a policy in relation to other states [10,11,12]. However, neither of these measures can explain the reasons why the extent or number of DUI laws adopted by each state varies. As an alternative to a single policy adoption, Stream [13] argued that more comprehensive policy solutions are adopted by more innovative states. In other words, the extent of adopted policies (for a particular policy problem) can be an appropriate measurement of innovativeness instead of considering the adoption of a single policy, when a state has adopted a “package” of policy options to achieve the policy goal. In some cases, later and less innovative adopters can adopt more comprehensive and efficient solutions than early and more innovative states. However, this pattern may occur because of their learning or mimicking of other more innovative states, and not due to their own state’s innovativeness. Nice [12], Haider-Markel [14], and Yu, et al. [15] used similar indices of comprehensiveness as dependent variables in their policy studies, while Mintrom and Vergari [16] did likewise in their article on school choice. Stream [13] constructed an additive scale of ten small group insurance market reform policies to measure the comprehensiveness of each state’s adoption of policies to correct group insurance market flaws. In order to understand which states address DUI laws more or less comprehensively, we employ as our dependent variable the comprehensiveness of a state’s adoptions of sixteen relevant DUI laws in our models, and select theory-relevant independent variables to explain why some states have actively adopted many DUI laws, while others have not (as is common in policy adoption research, we note that adoption does not mean it is fully implemented or enforced, but our research studies the comprehensiveness of DUI laws adopted, and not how well they are enforced).

## 3. Explanations of State’s Adoption of DUI Policies

In this section, we develop explanations of states’ adoptions of DUI laws with specific hypotheses based on both the policy innovation and diffusion literature, and also the traffic safety and DUI policy areas.

### 3.1. Innovativeness of Policy Decision Makers

#### 3.1.1. State Innovativeness as a General Trait of State Policy Decisions

Strategic choice theory argues that leaders of organizations do not merely react to external environmental changes but that they purposefully and strategically take actions to respond to societal or agency problems [17,18], and most of the research in strategic management proceeds from this assumption [19,20,21]. Likewise, state leaders’ adoption of new polices can be understood as a consequence of strategic decision-making and has a major influence on the process of policy adoption and diffusion from the perspective of strategic choice theory. Yet, mainstream policy innovation and diffusion studies have largely focused the patterns of diffusion on single policies and not integrated state innovativeness as a general trait of decision-makers (an exception is Boehmke and Skinner, [2]), in part because past studies have not found consistent support for high levels of innovativeness among states being consistent across all policies. However, in this study, we are examining the innovativeness of states that adopt more comprehensive DUI laws rather than fewer piecemeal policy choices. 

According to Walker’s [10] argument, policy innovation is defined as “a program or policy which is new to the states adopting it, no matter how old the program may be or how many other states may have adopted it”. He developed an index of policy innovativeness of each state by adding the rank order of states based on a state’s year of adoption of each of the 88 policies (which diffused across states from 1870 to 1966). His results indicated a modest level of state innovative consistency across the 88 policies and was criticized by Gray [11] as being too variable by policy area to combine into one index. Damanpour [22] noted that innovativeness of an organization must be measured based on multiple adoptions of several innovations. Since then the overall innovativeness index of a state has been developed by political scientists [11,23,24] and most recently by Boehmke and Skinner [2] using 180 state policies, with higher internal correlations. Boehmeke and Skinner [2] conclude “by using a rate score and by creating measures of uncertainty, we are able to statistically evaluate the original motivating question of whether states vary in their proclivity to innovate. Our analysis responds resoundingly in the affirmative.” Furthermore, they state “our data indicate a persistent and positive trend in innovativeness” displayed by the states over the time period we cover [2]. Considering the evidence that there are more innovative states in policy adoption, we expect that more innovative states are likely to adopt more DUI policies early compared to other states during a set period. 

DUI is a good policy area to study the correlation of why the extent of a state’s policy adoptions in one policy area vary substantially by the state’s innovativeness because each state’s single DUI law (among the sixteen we have identified) can be understood as an innovative approach to reduce drunk driving. If a state is more innovative, and thus, less risk averse to adopt new policies, we could expect that the state would be more likely to adopt more DUI laws than other states. Therefore, the first hypothesis related to state’s innovativeness is:

**Hypothesis** **1-1:** The higher the innovativeness score of a state government in general, the more likely it will adopt a higher number of DUI laws.

#### 3.1.2. State’s Innovativeness in Relevant Policy Areas for Traffic Safety

DUI laws are a key component of the broader policy area of traffic safety, especially driving safety. In addition to DUI laws, driving safety laws cover most of the other problematic driving behaviors that have been identified and outlawed, or required aggressive driving, child passenger safety, distracted driving, use of cell phones, seat belts, speed limits, etc. Theoretically, traffic safety laws could be viewed as a substitute for, or complement to, DUI policies (we thank a reviewer for this insightful comment). We argue that states are likely to view DUI policies as complements to broader traffic safety laws, thus states that are more innovative across the broad area of traffic safety laws are more likely to be more innovative in the narrower set of laws related to drink driving. Thus, we argue that state decision makers may consider bundles of policies in narrower and broader related policy areas, and strategically adopt more of the laws when traffic safety or drunk driving becomes a high state priority issue. Therefore, the second hypothesis is;

**Hypothesis** **1-2:** The higher the innovativeness score of a state government in traffic safety policies, the more likely that it will adopt a higher number of DUI laws.

#### 3.1.3. Organization Factors and Innovativeness

Even though an individual is the unit of analysis in Rogers’ original innovation-diffusion model, organizational theorists quickly adopted its important components to explain the adoption of new innovations at an organizational level [25]. Over time and research, organizational size, complexity (professionalism), and slack resources have been positively linked with organizational innovativeness [26,27], while large organizations tend to adopt more innovations than smaller ones [28,29,30]. Higher levels of organizational capacity and professionalism are positively associated with innovative agency and policy actions [31,32,33], and a higher level of slack resources enhances the organizational adoption of innovations, as more resources allow organizations to afford innovation, buffer failures, and cope with the costs of instituting innovations [27,34]. Thus, we follow in this tradition of using such variables in comparative state studies, recognizing these may be necessary but not sufficient conditions (we thank an anonymous reviewer for this point). Our contribution is to apply this logic to the level of state highway-related agencies, thus testing it in a new policy context.

**Hypothesis** **1-3:** *The larger the size of a state highway-related agency jobs, the more likely that it will adopt a greater number of DUI laws.*

**Hypothesis** **1-4:** *The higher the level of professionalism in a state highway-related agency, the more likely that it will adopt a higher number of DUI laws.*

**Hypothesis** **1-5:** *The better the state’s fiscal health (more slack resources), the more likely that it will adopt a greater number of DUI law.*

### 3.2. Diffusion through Bandwagon Effects and Relevant Controls

Scholars of innovation and diffusion have generally written that diffusion occurs through the mechanisms of learning, imitation (or mimicry), competition, or coercion [35], and each of these mechanisms assumes that decision makers have different motivations for adopting policies. Government A imitates government B when A adopts a policy adopted by B simply “in order to look like (B)” [36]. Imitation occurs because policymakers in A perceive B as worthy of emulation, prompting A to adopt any policy that B adopts independently of any evaluation of the character of the policy or its effectiveness [37,38,39]. Dimaggio and Powell discuss how agencies may imitate due to shared professional norms or pressure to confirm to well-regarded practices [40]. One form of learning or imitation diffusion has been called “bandwagon” diffusion, although it has primarily been used in studying management rather than policy diffusion.

Abrahamson and Rosenkopf’s definition of “bandwagon” diffusion is a process through which a management fad is spread across different systems or agencies, usually against rational efficiency theory, which distinguishes it from the learning diffusion process [6]. They argue that if the assessments of its outcome and effect are ambiguous, the technical, organizational, or strategic innovation is likely to be diffused in a bandwagon manner. The implication from this finding is that bandwagons may facilitate diffusion or rejection of innovations, regardless of their efficiencies; thus, both efficient but especially inefficient innovations may diffuse in a bandwagon pattern. Abrahamson and Rosenkopf delineated two types of bandwagon pressures: institutional bandwagon pressures and competitive bandwagon pressures [6]. Institutional bandwagon pressures occur when non-adopters are afraid to be different from the current, and often majority of, adopters. Thus, the institutional bandwagon pattern can be measured by the gap between the average number of all states’ adoptions and a certain state’s adoptions among DUI laws.

**Hypothesis** **2-1:** *As the gap between the average number of all states’ adoptions and a state’s adoptions of DUI policies increase over time, the state will be more likely to adopt more DUI laws.*

Competitive bandwagon effects occur because non-adopters worry about having below average performance if many adopters seem to enjoy a profit from their adoptions. Thus, competitive bandwagon effects can be seen as non-adopters’ concern that early movers will gain competitive advantage over them, even though it is uncertain whether early adopters really get a profit from their adoptions and early adopters’ policy choices would also be suited for non-adopters. Related to DUI policies, we posit that the gap between the average decrease of fatalities in the states with more DUI policies and the decrease of fatalities in a non-adopting state will create a competitive “bandwagon” pressure on the state decision makers.

**Hypothesis** **2-2:** *As the gap between fatalities in states with more DUI laws increases relative to states with fewer DUI laws, the state will be likely to adopt more DUI laws.*

We include a number of state-level control variables that might affect the perceived need for DUI laws: level of alcohol consumption, unemployment rate, and size of the population aged 15–24 years who are most likely to engage in alcohol abuse. Additionally, we include two types of state political ideology: citizen ideology, and government ideology of elected public officials, since many policies have a clear relationship to conservative or liberal political ideology.

## 4. Data and Method

We built a data set capturing the longitudinal and spatial aspects of states’ adoption of sixteen DUI laws. The panel data set is preferred in order to incorporate annual indicators of factors relevant to the states’ adoptions of DUI policies. The data set covers the years between 1990 and 2010 when these DUI policies were adopted. All independent variables are lagged one year because at minimum, one legislative session is necessary for a state legislature to decide to adopt a specific policy or law.

### 4.1. Dependent Variables

Our dependent variable is a sixteen-point additive scale, created by annually assigning points for the number of DUI policies each state has adopted. Table 1 identifies the state DUI policies included in the study and data source (among these laws, the federal government strongly encouraged states to adopt MLDA to 21, a BAC limit of 0.08, the Zero Tolerance Law, and the Open Contain Laws by forcing states to choose between passing these laws by a specific year and losing a portion of their federal highway construction funding. However, the adoptions of these laws are treated in the same way as the other law in this paper because the more innovative states had already adopted these laws prior to the federal government intervention. Also, there are substantial variations between states in the adoption years of these laws after the federal government intervention. Furthermore, in case of the open container law, only 43 states have enacted this law: Title of Law (Federal government intervention/the time limit): MLDA to 21 (1984/1988), BAC to 0.08 (2000/2004), the Zero Tolerance Law (1995/1998), and the Open Contain Laws (1988/NA)). If a state has adopted a DUI policy in year t, then a +1 is assigned to that state’s index for the year. In order to build a reliable scale on state DUI policy adoptions, the relationship between the individual DUI policy and the composite scores were tested and the value of the Cronbach Alpha is 0.7022 (See “Appendix A”). In summary, each state’s DUI policy index is a summated scale of dummy variables for whether a state has a specific policy among the sixteen DUI laws, with the dependent variable potentially ranging from 0 to 16. The actual range in the DV is from 10 to 15 laws adopted and amended (see Appendix B for each of the fifty states showing the pattern of DUI laws adopted).

### 4.2. Independent Variables

#### 4.2.1. State General Innovativeness

We use the measure of state innovativeness developed by Boehmke and Skinner in 2012 to capture overall state policy innovativeness [4]. They addressed the weakness of Walker’s original approach of innovation score by updating his measure with new data covering 189 different policies, using new methodological approaches, including the rate of innovation, and spatial auto-regression estimators. They used event history analysis (cross-sectional time series) as the basic logic of their innovativeness scores to solve the problem of right-censoring, which introduced bias in the innovation scores, and to measure state innovativeness changes over time. Their analysis compares states based on whether a state adopts a policy within a specific time period, rather than solely when a state adopts a policy. 

In their equation, Y_ikt_ is the zero in years of non-adoption and one in the year of adoption. After an adoption year, it is treated as missing in the following years. Thus, the numerator represents the cumulative number of state i’s adoptions in the year of t. K_it_ is the number of policies adopted by at least one state in year t; that is to say the denominator of this equation is the number of possible adoptions by state i at that time point.

(1) $$R_{it}=\;\frac{\sum_{i=1}^{K_{it}}Y_{ikt}}{K_{it}}$$

Furthermore, this measure can be used in calculating the changes in a state’s innovativeness over time from year T_0_ to T by adding up the total of a state’s adoptions and dividing by the number of total annual adoption opportunities during the time period. This logic is expressed in the following equation:

(2) $${\bar{R}}_{ij}=\;\frac{\sum_{t=T_{0}}^{T}\sum_{i=1}^{K_{it}}Y_{ikt}}{\sum_{t=T_{0}}^{T}K_{it}}$$

Thus, they conclude that the level of state innovativeness is reasonably estimated by how many policies the state adopts among possible adoptions within specific time periods, as well as over the entire 1804–2008 period.

#### 4.2.2. State Issue Specific Innovativeness 

We use the measure of state innovativeness developed by Boehmke and Skinner in 2012 to capture overall state policy innovativeness. They addressed the weakness of Walker’s original approach of innovation. However, as mentioned previously, the general innovativeness of the state is not necessarily the same as the issue-specific innovativeness of a state [11]. To measure the issue-specific innovativeness of a state, first of all, data for the policies relevant to traffic safety are gathered. To measure the overall traffic safety innovativeness, DUI laws should be included because reducing drunk driving is one of the core sub-policy areas in traffic safety policy. However, our interest in this paper is the influence of state innovativeness in other traffic safety areas on the comprehensiveness of a state’s policy adoptions of DUI laws, thus, we do not include state DUI laws in measuring state traffic safety innovativeness here. ”Issue specific innovativeness” as measured in this paper means “State traffic safety innovativeness without including DUI policies”. A total of ten policies and programs related to traffic safety are included in the issue specific innovativeness (ten policies and programs related to traffic safety: Aggressive driving law, Auto-enforcement law related to speed, Auto-enforcement law related to red-light camera, Graduated Driver Licensing (GDL), License law for old drivers, Seat belt law, the ban of hand-held cellphone law, the ban of mobile texting law, and Work-zone safety law). State innovativeness related to traffic safety is recalculated using these ten laws, using the same metric as was performed for the general state innovativeness by Boehmke and Skinner [4]. More specifically, a state’s innovativeness in traffic safety policy is estimated by how many traffic safety policies the state has adopted among possible adoptions in a specific year. 

**Figure 1:**
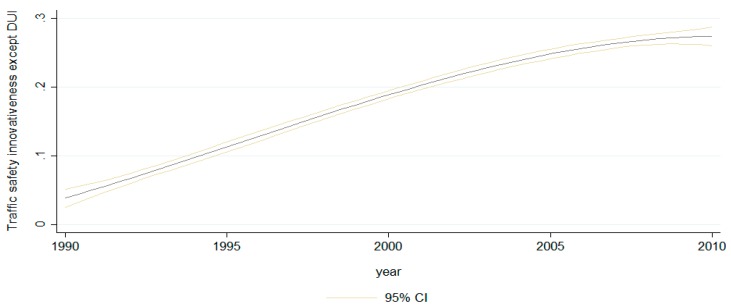
Traffic safety innovativeness, except drunk driving policy area, 1990–2010.

**Figure 2:**
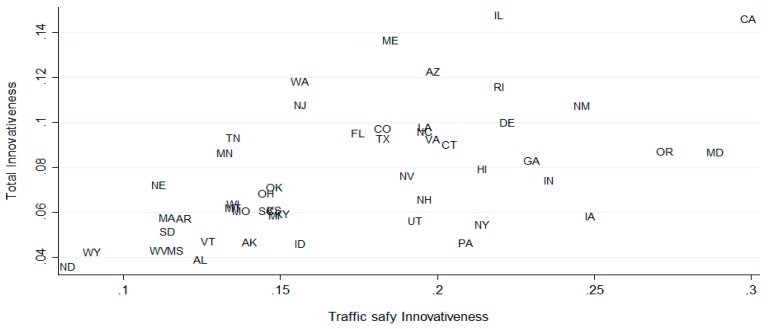
General state innovativeness versus traffic safety innovativeness, except drunk driving policy area, 1990–2010.

Figure 1 shows the pattern of change in traffic safety innovativeness across all states from 1990 to 2010. In this graph, state traffic safety innovativeness outside DUI policies has gradually increased (each state’s traffic safety innovativeness outside DUI policy are presented in Appendix C). Figure 2 is the scatterplot of state general innovativeness and traffic safety innovativeness; the correlation between these is 0.572 at a 99% significance level from 1990 to 2010, demonstrating a moderate positive correlation between traffic safety innovativeness and general state innovativeness (the results of the multi-collinearity test are presented in Appendix D).

#### 4.2.3. Organizational Innovativeness

Organizational factors often associated with organizational innovativeness include organizational size, slack resources, and professionalism [26,27]. To measure the organizational size, we use the number of state government employees per ten miles of highway in a state in jobs that relate to traffic safety. The professionalism for a state government is measured by the average payroll of the state employees in highway jobs (this measurement is similar to legislative professionalism measured based on state legislator salaries [44,45,46,47]). More specifically, total payroll in highway work-related jobs is divided by the total number of employees in highway jobs for measuring state professionalism in traffic-related fields (Annual Survey of Public Employment and Payroll was not done for 1996 because the base reporting period for these changed from October to March. This change was introduced with the 1997 Census of Governments. Therefore, the same data as 1997 is used for 1996 in this analysis). The level of slack resources of a state government is represented by the state’s fiscal health, which is measured by the ratio of a state’s total-revenue minus total spending to total spending. These data are obtained from “Government Employment and Payroll” and “Statistical Abstract of the United States” from 1990 to 2010, published by the United States census bureau. 

#### 4.2.4. Bandwagon Effects and Relevant Controls

Two types of bandwagon effects are tested in our models. The institutional bandwagon can be measured by the difference between the national average of states’ adoptions and a state’s adoptions among DUI laws, since institutional bandwagon pressures are created by non-adopters’ fear of being seen as different from other states. Competitive bandwagon effects occur when decision makers believe the average performance of most policy adopters is better than the performance of non-adopters. Therefore, we measure competitive bandwagon effects as the average of alcohol-related fatalities in a specific state minus the average of alcohol-related fatalities in states that have adopted more DUI policies than the specific state. The data of alcohol-related fatalities are obtained from NHTSA annual reports from 1990 to 2010.

Citizen ideology is measured using the well-established measures created by Berry, Ringquist, Fording, and Hanson, which is the average position of the active constituents on a continuum from liberal (1) to conservative (0) in a state, while state government ideology is the ideological mean score produced based on the distribution of power among the state’s primary elected public officials—the governor and the major party representatives in the state legislature [48]. We use annual alcohol consumption in a state as an alcohol-related control variable, measured in gallons of ethanol from all alcoholic beverages consumed annually per capita; this data came from the alcohol consumption trends for 1977–2012 [49]. The data on the state unemployment rates and percentage for population aged 15 to 24 (natural log) are taken from “Statistical Abstract of the United States 1990–2010” [50]. Table 2 lists the names and definitions of variables used in our analysis.

### 4.3. Method

To investigate the determinants of state adoptions of DUI laws across states, the statistical estimation technique should ideally be capable of analyzing temporal and spatial patterns of policy adoptions. Therefore, panel analysis is conducted in this paper, because this method is suited for studying all 50 states (same units) for twenty years (multiple times); this study uses 998 observations, 50 states, and 20 years period (from 1990 to 2010). The STATA 14.2 statistics software program (StataCorp, College Station, TX, USA) was used to conduct our analyses. Also, a statistical test (F test) supports that panel analysis is better than pooled OLS (Ordinary Least Squares) (*p* = 0.00). Concerning the selection between fixed effect and random effect models, the Hausman Test shows that the fixed effect model is more relevant and significant than the random effect model (Prob > chi2 = 0.00). 

However, “heteroscedasticity” and other statistical problems can arise when using panel analysis. Relevant tests, namely Wooldridge, Pesaran, and Modified Wald tests, were conducted; autocorrelation, heteroscedasticity, and cross-sectional dependence were detected in our initial models as a result of these tests (Wooldridge test for autocorrelation in panel data (Prob > F = 0.0000), Pesaran’s test of cross-sectional independence (Pr = 0.0000), and Modified Wald test for group-wise heteroskedasticity in fixed effect regression model (Prob > chi2 = 0.0000)). To deal with these statistical issues, we applied fixed-effect regression with Driscoll and Kraay standard errors to our panel analysis, because the error structure of Driscoll and Kraay assumes that there are autocorrelations, possible cross-sectional dependency, and heteroscedasticity in panel data [51]. 

## 5. Results

Overall, results of the statistical analysis (shown in Table 3) provide considerable support for most (four out of seven) of our hypotheses. State policy adoptions of DUI laws are highly associated with enactment of innovative traffic safety policies (Hypothesis 2) but are unrelated to the overall policy innovativeness of a state (Hypothesis 1). The comprehensiveness of a state’s DUI policy adoption will increase by 6.048, whenever the rate of the state’s traffic safety innovativeness (omitting DUI policies) increases by one, holding all other variables constant (*p* < 0.000).

Two of our three hypotheses related to organizational innovation received statistical support at a significance level of 0.001. Adoption of the DUI policy bundle comprehensiveness is highly associated with size (Hypothesis 3) and professionalism (Hypothesis 4) of the state’s government but has no relationship to the state’s fiscal health (Hypothesis 5, which we tested as a proxy for slack resources). Our results demonstrate that when the number of state government employees per 10 miles of highway in highway jobs increases by one, the comprehensiveness of state adoptions against drunk driving increases by 1.074 (*p* < 0.000). Based on this, we find confirming support for Hypothesis 1-3—more DUI policies are likely to be adopted by a state with a larger size of employees in highway work-related jobs. Our results also confirm Hypothesis 1-4—a state with a higher level of professionalism is more likely to adopt comprehensive DUI laws. Specifically, as the average payroll of state employees in highway jobs increases by one thousand dollars, the comprehensiveness of a state’s adoption of DUI policies will increase by 1.114, when all other variables are held constant (*p* < 0.000). These results can be interpreted as indicating that organizational size and professionalism have a strong and significant relation to the comprehensiveness of state adoptions in the DUI policy bundle. 

Neither citizen or government ideology were related to the comprehensiveness of DUI policy adoptions, suggesting that policies related to reducing drunk driving are nonpartisan in the states, and viewed as nonpartisan by both regular citizens as well as elected legislators and governors. While DUI policies have been highly salient and a political issue for decades, the parties are generally aligned in how to address drunk driving. Comprehensiveness in DUI policies was highly related to alcohol consumption, suggesting that this indicator of an alcohol driving problem was an indicator of policy need. Interestingly, the control variable for the size of the younger (15–24) population in the state was highly negatively-related to the innovativeness of the state in adopting DUI polices, which was opposite to expectations. Unemployment rates were unrelated to DUI comprehensiveness.

Finally, the institutional bandwagon measure was highly positively-related to adoption of DUI policy bundles, supporting Hypothesis 2-1, while the competitive bandwagon measure showed no strong relationship with DUI policy comprehensiveness. These findings together suggest that states are looking to keep up with their “sister” states by adopting more policies as more states have already enacted them (furthermore, considering the relevance of explanatory variables in the model to the dependent variables, the institutional bandwagon pressure is more relevant than other explanations because its *t*-value (34.98) is much higher than any other independent variables. Based on these results, the institutional bandwagon effect can be regarded as one of the persuasive mechanisms to explain the diffusion pattern of states’ adoptions in the DUI policy area).

## 6. Conclusions 

This study examines the factors related to the comprehensiveness of the package of state DUI laws states enact, focusing on the impact of the state’s overall policy innovativeness, and the innovativeness of policies related to highway safety. We also test common factors found in innovation research—size, professionalism, and wealth—to tie this study to the broader innovation world of research. Finally, we test for the competing processes, called institutional and competitive bandwagon effects, on states related to pressures to adopt more policies as more states enact the DUI policies. This is a type of diffusion that is rarely studied in policy diffusion, with neighboring state influence being the most common type of diffusion pressure studied. Sixteen state-level DUI laws are used to measure the comprehensiveness of a state’s package of DUI policies. Our study uses a panel design of policies from the fifty states covering the period from 1990 to 2010. Table 4 summarizes our findings according to the hypotheses we test.

We find that states that are more innovative in traffic safety policies, not in general policy innovativeness, are more likely to adopt a higher number of DUI laws. Few studies have examined if states adopt a comprehensive package of laws in a policy subfield or adopt policies that could be considered substitutes or partial coverage laws. This study shows a clear pattern that innovative states in the traffic safety policy arena have adopted more laws rather than fewer among the sixteen laws that constitute our dependent variable index related to DUI. Also, we find that states with a higher level of state-highway agency professionalism and a larger number of staff in the state highway agency are more likely to be comprehensively innovative in traffic safety policies. The size and professional factors have been found in many studies on state policy innovation to be associated with innovative states [35]. 

Furthermore, we introduce the concept of diffusion by bandwagon effect, which has mainly been used in management innovation and diffusion studies; our results show the institutional bandwagon effect is a primary explanatory variable in our model associated with the comprehensiveness of state DUI polices. Various reasons could explain this result. Perhaps social pressure to do something to address rising levels of drunk and distracted driving accidents and fatalities led to states adopting more policies, regardless of whether they are effective or not. Perhaps states with more comprehensive DUI laws received good media coverage or were viewed as being leaders in the constant battle to reduce DUI fatalities and accidents. Our results only support the hypothesis for institutional bandwagon (Hypothesis 2-1); results were neutral for the competitive bandwagon hypothesis (Hypothesis 2-2). In bandwagon diffusion, states adopt a policy through mimicking or isomorphic effects when that policy seems popular, has good intentions, and has little to no political opposition. 

Interestingly, we find that there is not any strong and significant relation between prevalent political ideology in a state and the comprehensiveness of the state’s DUI policy adoptions. The current empirical research by Macinko and Silver shows a similar result—that the political makeup of a state government is not the most influential predictor in alcohol policy adoptions in the state [52]. This demonstrates that state’s policy adoptions against drunk driving are not the product of partisan politics. Thus, the accumulated findings indicate that a state’s adoptions of DUI policies are best understood as nonpolitical management approaches or politically popular policies to control drunk driving in the state. By theory, the bandwagon effect occurs due to a state’s concern about institutional dissimilarity to other states, and not as a result of comparing its below average performance to other states that have more alcohol-impaired driving policies. 

One of the contributions of this paper to the innovation and diffusion literature is our use of a 16-policy index for the dependent variable, and our focus on innovativeness in state highway safety policies, as well as in overall policy innovativeness. We consider issue specific innovativeness in the traffic safety policy area as a narrowly-defined or alternative measure of overall state innovativeness in this study, to assess the broader impact of state policy impact on the adoption of a bundle of DUI policies. Our findings support older arguments that states are not likely to be innovative in all policy areas [11]. While it is still debated among state innovation scholars whether an overall state innovativeness pattern exists, Boehmke and Skinner’s [4] index of 180 policies covers the time period of 1912 through 2008, much longer than the 20 year period DUI policies cover. Rather, our results demonstrate that states in related narrower policy areas may have tastes for more comprehensive policy bundles within specific policy areas, such as highway safety policies in this study. 

In addition, the size, professionalism, and slack resources of a state are considered as components of the state’s organizational innovativeness in this study, following many studies of innovation and diffusion results. While the concept of state innovativeness focuses on the decision-making stage in the public policy process, organizational innovativeness puts more weight on the organizational capacity for implementing relevant policies. Our results suggest that if a state does not have enough capacity to implement a policy, the state may be less likely to adopt more policies. We find that larger states and states with a higher level of government professionalism are more likely to be comprehensively innovative in the adoption of DUI policies. 

In summary, it can be said that the comprehensiveness of state DUI policy adoption is decided by a state’s innovativeness in the traffic safety policy area, organizational size and professionalism in a state government relevant to traffic safety, and bandwagon effect created by institutional dissimilarity across states under the constraints represented by internal characteristics of a state.

There are a number of limitations in this paper. Historically, nonprofit organizations represented by MADD (Mothers Against Drunk Driving) have a huge impact on the expansion of alcohol-impaired driving policies. Because of the limitations of this dataset, the influence of interest groups is not tested as well. Also, our findings are not conclusive, since they are based on one policy area. 

However, this finding and pattern has not been demonstrated before in comparative state policy studies. Thus, as a meaningful next step, it would be good to examine the influence of state innovativeness in other similar policy areas, such as health regulations on tobacco, marijuana, or other harmful practices. Furthermore, it could be worthy to examine different patterns in the influence of state innovativeness dependent on policy typology, such as moral and economic factors, among others.

## Figures and Tables

**Table 1:** State DUI (Driving Under the Influence) laws included in analysis.

State Laws	Data Source
False id law	Alcohol policy information system (APIS) [41]. Statewide Availability Data System II: 1933–2003 (Pacific Institute for Research and Evaluation, Prevention Research Center) [42]. Digest of State U.S. Alcohol-Highway Safety Related Legislation from 1990 to 2010 [43].
Zero tolerance law	
0.10 BAC (Blood Alcohol Concentration) law	
0.08 BAC (Blood Alcohol Concentration) law	
High BAC (Blood Alcohol Concentration) law	
Mandatory use of an interlock device (first offender)	
Mandatory use of an interlock device (repeated offender)	
Alcohol exclusion law	
Administration revocation law	
Dram shop law	
Open container law	
Anti-consumption law	
Keg Registration	
Sobriety check point law	
Drug Per Se Law	
Drug Evaluation and Classification Program (DECP)	

**Table 2:** Variable descriptions: State DUI policies.

Theoretical Explanation	Variable		Description
**Innovativeness Models**	State General Innovativeness		State General Innovativeness calculated by Boehmke and Skinner (2012) [4]
	Traffic Safety Innovativeness (except DUI policy area)		State Traffic Safety Innovativeness calculated by author in the same way as Boehmke and Skinner (2012) [4]
	Organizational State Innovativeness	Size of a state highway-related agency jobs	The number of state government highway job-related employees per 10 miles of highway in a state
		Professionalism in a state highway-related agency	Average payroll of highway work-related employees (total payroll in highway work-related is divided by the total number of employees in highway jobs)
		The Level of Resource Slack	Fiscal health (the ratio of total-state-revenue minus total-state’s spending to total spending)
Bandwagon Models	Institutional Bandwagon		Institutional dissimilarity (the gap between the national average of states’ adoptions and a state’s adoptions among DUI laws)
	Competitive Bandwagon		Below performance compared to other states with more DUI policies (the gap between the average of alcohol-related fatalities in a state and the average of alcohol-related fatalities in states with more DUI policies than the state)
Relevant Controls	Political Ideology	Citizen Ideology	Citizen and Government Ideology measurement (a higher score indicates a more liberal: 1–100 scale)
		Government Ideology	
	Alcohol Consumption		Gallons of ethanol per capita in a state
	Younger Population		Natural logarithm of younger population aged 15 to 24 in a state
	Unemployment Rate		A percent is calculated by dividing the number of unemployed persons by the number of persons in the labor force

**Table 3:** Fixed effect estimation with Driscoll and Kraay standard errors.

Independent Variables	Fixed Effect with Driscoll-Kraay (Robust Standard Error)
State General Innovativeness	−0.464
	(0.405)
Traffic Safety Innovativeness (except DUI policy area)	6.048 **
	(0.890)
The size of a state highway-related agency jobs (Organizational Innovativeness)	1.074 **
	(0.371)
The Professionalism in a state highway-related agency (Organizational Innovativeness)	1.144 **
	(0.109)
The Level of Resource Slack of State government (Organizational Innovativeness: Fiscal Health)	−0.004
	(0.308)
Institutional Bandwagon effect	1.020 **
	(0.029)
Competitive Bandwagon effect	−0.159
	(0.415)
Political Liberalism (Citizen)	0.000
	(0.002)
Political Liberalism (Government)	−0.002
	(0.002)
Alcohol Consumption	2.068 **
	(0.365)
Younger Population	−0.482 **
	(0.048)
Unemployment Rate	4.414
	(3.229)
Constant	13.619 **
	(1.370)
Number of cases	998
Number of groups	50
R^2^ (within)	0.8969
F-test	14,774.66 **

Note: * *p* < 0.05, ** *p* < 0.01.

**Table 4:** Comparison of hypothesis predictions and results.

Hypothesis	Predicted Direction	Findings
H1-1: General policy innovativeness	Positive	No Effect
H1-2: Highway safety policy innovativeness	Positive	Positive (0.001)
H1-3: Size of government	Positive	Positive (0.001)
H1-4: Level of professionalism (capacity)	Positive	Positive (0.001)
H1-5: Government Fiscal Health (slack resources)	Positive	No Effect
H2-1: Institutional Bandwagon	Positive	Positive (0.001)
H2-2: Competitive Bandwagon	Negative	Negative but no effect

**Figure A1:**
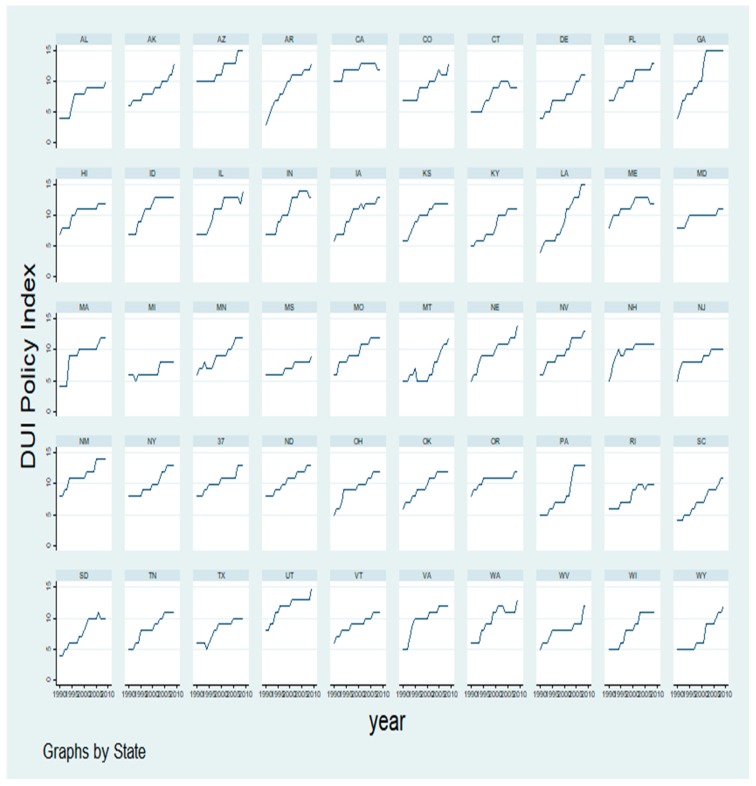
Each State’s DUI Policy Index—The Comprehensiveness of DUI Policies of a State.

**Figure A2:**
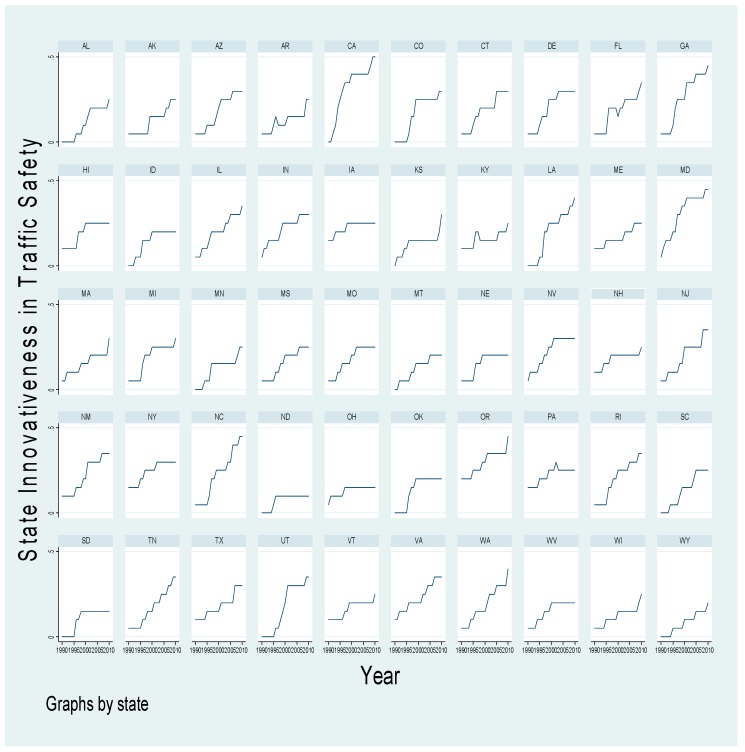
Each state’s traffic safety innovativeness outside DUI.

## Appendix A: Reliability Test for Dependent Variables

To build a scale on DUI policy adoptions, the relationship between the individual policy and the summated score is tested. The internal consistency of a scale is measured by Cronbach’s alpha (1951) and a widely accepted rule of thumb related to Cronbach’s alpha is that it should be at least 0.70 to demonstrate the internal consistency of a scale. The alpha coefficient reported in Table for the comprehensiveness scale of DUI policies below is acceptable at 0.7022 (standardized).

**Table A1:** Item Total Statistics for the Comprehensiveness Scale of DUI policies.

Scale	Scale Mean If Item Deleted	Alpha
False id law	8.693	0.6963
Zero tolerance law	8.831	0.6689
0.10 BAC law	8.659	0.7056
0.08 BAC law	9.047	0.6516
High BAC law	9.238	0.6606
Mandatory use of an interlock device (first offender)	9.593	0.6911
Mandatory use of an interlock device (repeated offender)	9.520	0.6763
Alcohol exclusion law	8.889	0.6879
Administration revocation law	8.836	0.6923
Dram shop law	8.773	0.7224
Open container law	8.943	0.6861
Anti-consumption law	8.815	0.7051
Keg Registration	9.253	0.6843
Sobriety check point law	8.913	0.7140
Drug Per Se Law	9.489	0.6859
Drug Evaluation and Classification Program (DECP)	8.843	0.6744
Test Scale		0.7022

## Appendix D: Multi Collinearity Test for Independent Variables

**Table A2:** Multi Collinearity Test for Independent Variables.

Variables	VIF	1/VIF
Traffic Safety Innovativeness (except DUI policy area)	2.08	0.4816
Political Liberalism (Government)	1.81	0.5538
Political Liberalism (Citizen)	1.79	0.5597
The Professionalism of State government (Organizational Innovativeness)	1.74	0.5750
Younger Population	1.51	0.6623
Unemployment Rate	1.39	0.7201
The Level of Resource Slack of State government (Organizational Innovativeness: Fiscal Health)	1.34	0.7435
State General Innovativeness	1.25	0.8026
Alcohol Consumption	1.23	0.8135
Competitive Bandwagon effect	1.15	0.8719
Institutional Bandwagon effect	1.11	0.8988
The Size of State government (Organizational Innovativeness)	1.07	0.9311
Mean VIF (Variance Inflation Factor)	1.45	

## References

[1] National Highway Traffic Safety Administration (2014). Traffic Safety Facts 2013.

[2] Mounce N.H., Pendleton O.J. (1992). The relationship between blood alcohol concentration and crash responsibility for fatally injured drivers. Accid. Anal. Prev..

[3] Boushey G. (2010). Policy Diffusion Dynamics in America.

[4] Boehmke F.J., Skinner P. (2012). State policy innovativeness revisited. State Polit. Policy Q..

[5] Nicholson-Crotty S.C., Woods N.D., Bowman A.O., Karch A. (2014). Policy Innovativeness and Interstate Compacts. Policy Stud. J..

[6] Abrahamson E., Rosenkopf L. (1993). Institutional and competitive bandwagons: Using mathematical modeling as a tool to explore innovation diffusion. Acad. Manag. Rev..

[7] Kennedy M.T., Fiss P.C. (2009). Institutionalization, framing, and diffusion: The logic of TQM adoption and implementation decisions among U.S. hospitals. Acad. Manag. J..

[8] Lee J.Y., Chan K.C. (2003). Assessing the operations innovation bandwagon effect: A market perspective on the returns. J. Manag. Iss..

[9] Rosenkopf L., Abrahamson E. (1999). Modeling reputational and informational influences in threshold models of bandwagon innovation diffusion. Comput. Math. Org. Theory.

[10] Walker J.L. (1969). The diffusion of innovations among the American states. Am. Polit. Sci. Rev..

[11] Gray V. (1973). Innovation in the states: A diffusion study. Am. Polit. Sci. Rev..

[12] Nice D.C. (1994). Policy Innovation in State Government.

[13] Stream C. (1999). Health reform in the states: A model of state small group health insurance market reforms. Polit. Res. Q..

[14] Haider-Markel D.P. (1998). The politics of social regulatory policy: State and federal hate crime policy and implementation effort. Polit. Res. Q..

[15] Yu J., Jennings E., Butler J. (2018). Lobbying, learning and policy reinvention: An examination of the American states’ drunk driving laws. J. Public Policy.

[16] Mintrom M., Vergari S. (1997). Charter schools as a state policy innovation: Assessing recent developments. State Local Govern. Rev..

[17] Child J. (1972). Organizational structure, environment and performance: The role of strategic choice. Sociology.

[18] Dougherty D., Heller T. (1994). The illegitimacy of successful product innovation in established firms. Org. Sci..

[19] Subramanian A. (1996). Innovativeness: Redefining the concept. J. Eng. Tech. Manag..

[20] Bryson J.M. (2018). Strategic Planning for Public and Nonprofit Organizations.

[21] Bryson J.M., Berry F.S., Yang K. (2010). The State of Public Strategic Management Research: A Selective Literature Review and Set of Future Directions. Am. Rev. Public Admin..

[22] Damanpour F. (1987). The adoption of technological, administrative, and ancillary innovations: Impact of organizational factors. J. Manag..

[23] Canon B.C., Baum L. (1981). Patterns of Adoption of Tort Law Innovations: An Application of Diffusion Theory to Judicial Doctrines. Am. Polit. Sci. Rev..

[24] Savage R.L. (1985). When a Policy’s Time Has Come: Cases of Rapid Policy Diffusion 1983–1984. Publius.

[25] Berry F.S., Berry W.D. (1990). State Lottery Adoptions as Policy Innovations: An Event History Analysis. Am. Polit. Sci. Rev..

[26] Burns T., Stalker G.M. (1961). The Management of Innovation.

[27] Rogers E.M. (1995). Diffusion of Innovations.

[28] Kimberly J.R., Evanisko M.J. (1981). Organizational innovation: The influence of individual, organizational, and contextual factors on hospital adoption of technological and administrative innovations. Acad. Manag. J..

[29] Damanpour F. (1992). Organizational size and innovation. Org. Stud..

[30] Walker R.M. (2006). Innovation type and diffusion: An empirical analysis of local government. Public Admin..

[31] Pierce J.L., Delbecq A.L. (1977). Organization structure, individual attitudes and innovation. Acad. Manag. Rev..

[32] Damanpour F. (1991). Organizational innovation: A meta-analysis of effects of determinants and moderators. Acad. Manag. J..

[33] Kwon M., Berry F.S., Feiock R.C. (2009). Understanding the Adoption and Timing of Economic Development Strategies in U.S. Cities Using Innovation and Institutional Analysis. J. Public Admin. Res. Theory.

[34] Rosner M.M. (1968). Economic determinants of organizational innovation. Admin. Sci. Q..

[35] Berry F.S., Berry W.D., Weible M.C., Sabatier P. (2018). Innovation and Diffusion Models in Policy Research. Theories of the Policy Process.

[36] Shipan C.R., Volden C. (2008). The Mechanisms of Policy Diffusion. Am. J. Polit. Sci..

[37] Simmons B.A., Dobbin F., Garrett G. (2006). Introduction: The International Diffusion of Liberalism. Int. Org..

[38] Meseguer C. (2006). Learning and Economic Policy Choices. Eur. J. Polit. Econ..

[39] Karch A. (2007). Emerging Issues and Future Directions in State Policy Diffusion Research. State Polit. Policy Q..

[40] DiMaggio P., Powell W.W. (1983). The iron cage revisited: Collective rationality and institutional isomorphism in organizational fields. Am. Sociol. Rev..

[41] Alcohol Policy Information System (APIS) APIS Policy Topics. National Institute of Alcohol Abuse and Alcoholism. https://alcoholpolicy.niaaa.nih.gov/APIS_Policy_Topics.html.

[42] Ponicki W.R. (2004). Statewide Availability Data System II: 1933–2003.

[43] National Highway Traffic Safety Administration (1990–2010). Digest of State US. Alcohol-Highway Safety Related Legislation 8–25.

[44] Ferraz C., Finan F. (2009). Motivating Politicians: The Impacts of Monetary Incentives on Quality and Performance.

[45] King J.D. (2000). Changes in professionalism in U.S. state legislatures. Legisl. Stud. Q..

[46] Squire P. (1992). The theory of legislative institutionalization and the California assembly. J. Polit..

[47] Eliassen K.A., Pedersen M.N. (1978). Professionalization of legislatures: Long-term change in political recruitment in Denmark and Norway. Comp. Stud. Soc. Hist..

[48] Berry W.D., Ringquist E.J., Fording R.C., Hanson R.L. (1998). Measuring citizen and government ideology in the American states, 1960–1993. Am. J. Polit. Sci..

[49] LaVallee R.A., Kim T., Yi H. (2014). Surveillance Report# 98: Apparent Per Capita Alcohol Consumption: National, State, and Regional Trends, 1977–2012.

[50] U.S. Census Bureau (2010). Statistical Abstract of the United States.

[51] Hoechle D. (2007). Robust standard errors for panel regressions with cross-sectional dependence. Stata J..

[52] Macinko J., Silver D. (2015). Diffusion of impaired driving laws among US states. Am. J. Public Health.

